# Assessing handwriting task difficulty levels through kinematic features: a deep-learning approach

**DOI:** 10.3389/frobt.2023.1193388

**Published:** 2023-09-13

**Authors:** Vahan Babushkin, Haneen Alsuradi, Muhammad Hassan Jamil, Muhamed Osman Al-Khalil, Mohamad Eid

**Affiliations:** ^1^ Applied Interactive Multimedia Lab, Engineering Division, New York University Abu Dhabi, Abu Dhabi, United Arab Emirates; ^2^ Tandon School of Engineering, New York University, New York, NY, United States; ^3^ Arabic Studies Program, New York University Abu Dhabi, Abu Dhabi, United Arab Emirates

**Keywords:** artificial neural networks, deep learning, learning from demonstration, machine learning, sensorimotor learning

## Abstract

**Introduction:** Handwriting is a complex task that requires coordination of motor, sensory, cognitive, memory, and linguistic skills to master. The extent these processes are involved depends on the complexity of the handwriting task. Evaluating the difficulty of a handwriting task is a challenging problem since it relies on subjective judgment of experts.

**Methods:** In this paper, we propose a machine learning approach for evaluating the difficulty level of handwriting tasks. We propose two convolutional neural network (CNN) models for single- and multilabel classification where single-label classification is based on the mean of expert evaluation while the multilabel classification predicts the distribution of experts’ assessment. The models are trained with a dataset containing 117 spatio-temporal features from the stylus and hand kinematics, which are recorded for all letters of the Arabic alphabet.

**Results:** While single- and multilabel classification models achieve decent accuracy (96% and 88% respectively) using all features, the hand kinematics features do not significantly influence the performance of the models.

**Discussion:** The proposed models are capable of extracting meaningful features from the handwriting samples and predicting their difficulty levels accurately. The proposed approach has the potential to be used to personalize handwriting learning tools and provide automatic evaluation of the quality of handwriting.

## 1 Introduction

Handwriting is a complex sensorimotor skill that combines sensory, cognitive, and motor processes to master. Forming legible handwriting requires a coordinated movement of the hand, arm and fingers while continuously evaluating the visual (formed letter) and the haptic (pressure from the pen) feedback received towards adjusting the trajectory, alignment and force of writing. The ability of children to produce fluent and readable handwriting is important for educational development, achievements in school, and self-esteem (Chang and Yu, 2014). The advances in assistive technologies, along with a better understanding of the difficulty of handwriting, led to new and innovative methods of handwriting development/evaluation (Maor et al., 2011) and rehabilitation (Fancher et al., 2018). With the availability of smart devices (such as touch devices and hand tracking technologies), the analysis of handwriting has reached new heights, such as handwriting evaluation (Rosenblum et al., 2003), detection/prediction of handwriting difficulties (Drotár and Dobeš, 2020), and novel remediation/intervention methods (Fancher et al., 2018).

The transformation from traditional teaching methods to technology-enhanced learning paved the way for exploring personalized learning paradigms (Jenkins et al., 2016). In fact, research has found that personalizing the learning task for individual learners improves motivation, attitude, and learning (Corbalan et al., 2006). A fully personalized handwriting learning system would involve a mechanism to evaluate the quality of handwriting as a sensorimotor skill and provide a learning task with an appropriate level of difficulty. Giving learners a learning task that is too difficult or too easy can hinder the learning process and cause a downgrade (Curto et al., 2015). Traditionally, the difficulty level of a handwriting task is evaluated through expert assessment using task-specific performance measures. Researchers have developed known metrics for evaluating the difficulty of handwriting tasks, including the size and complexity of the shape of the handwriting task, the number and sequence length of stroke, and the quality of sensorimotor skill of the writer (Danna and Velay, 2015). Understanding the difficulty level of a handwriting task is a challenge due to the high subjectivity/diversity among experts’ judgments. Furthermore, manually selecting a handwriting task for an appropriate level of difficulty is inefficient. Besides, automatically evaluating the difficulty level of a handwriting task is ill-defined. By using tablets and hand motion tracking, various handwriting features can be measured and analyzed, e.g., pressure, handwriting speed, in-air movement, stops and lifting of a pen, text shape, and time needed to complete the task, to evaluate the difficulty of a handwriting task. With the availability of data, it is tempting to utilize machine and/or deep learning to automatically classify the difficulty level of a handwriting task.

Machine learning has been used to detect if a child suffers from writing disabilities by using the kinematics of handwriting and questionnaires data (Pagliarini et al., 2017). Recent studies demonstrate the utilization of machine learning methods to diagnose dysgraphia (Mekyska et al., 2016; Asselborn et al., 2018; Asselborn et al., 2020; Dui et al., 2020). For instance, a Random Forest model was developed to identify dysgraphic children; the study included 54 third-grade children and used a 10-item questionnaire for Hebrew handwriting proficiency (HPSQ) (Rosenblum, 2008) to identify poor writing. Another study used commodity tablets and proposed a tool to diagnose dysgraphia (Asselborn et al., 2018); data from 298 children, including 56 with dysgraphia, were labeled using the Concise Assessment Scale for Children’s Handwriting (Beknopte beoordelingsmethode voor kinderhand-schriften, BHK) test and utilized it to train a Random Forest classifier to detect dysgraphia. Results demonstrated that the proposed model has comparable accuracy to experts. A subsequent study proposed a serious games tablet application for handwriting skill screening at the preliteracy stage (Dui et al., 2020). Instead of a global score based on the BHK test, the authors in (Asselborn et al., 2020) proposed a detailed profile that incorporates the kinematics, pressure, pen tilt and static features (such as the letter shape) in the evaluation of handwriting skills. Based on this evaluation, personalized remediation tasks that are very specific to the individual child’s needs can be developed. To our knowledge, no study examined both stylus and hand kinematics to evaluate the difficulty level of handwriting tasks.

This paper presents a data-driven model to provide an unbiased evaluation of the difficulty of the handwriting task, which in turn facilitates the personalized acquisition of the handwriting skill. The system will also be helpful for evaluating the quality of handwriting and informing the simulation of sensorimotor skills. Using the developed model, we examine prominent sensorimotor features based on kinematics that are found in both the hand (such as hand orientation and grip characteristics) and the stylus (including stylus orientation, pressure, and temporal characteristics). The proposed model is evaluated with data collected from five handwriting experts who completed the handwriting of all Arabic alphabet letters. We made the choice of using Arabic orthography as it captures a wider range of difficulty levels in handwriting (Naz et al., 2014), particularly due to its cursive nature, complex ligatures, and numerosity of topological features (Kacem et al., 2012). The model is expected to learn features that could deem a task difficult/easy. This task could go beyond the 28 letter of the Arabic alphabet such as letters-like shapes as well as word segments.

Two models have been considered–the one learning from the aggregated opinion of all experts about the difficulty of a given handwriting task, and the other learning the distribution of experts’ estimates of the difficulty level of a given handwriting task. While the first model can tell in average what difficulty level will be assigned to the handwriting task, the second model can explicitly predict how many experts estimate the handwriting task of a particular difficulty level (in order to capture the subjectivity/diversity of expert assessment of the handwriting difficulty level). Furthermore, Shapley values are applied to identify features that are prominent for the evaluation of the difficulty of handwriting.

## 2 Proposed approach

### 2.1 Experimental setup

**FIGURE 1:**
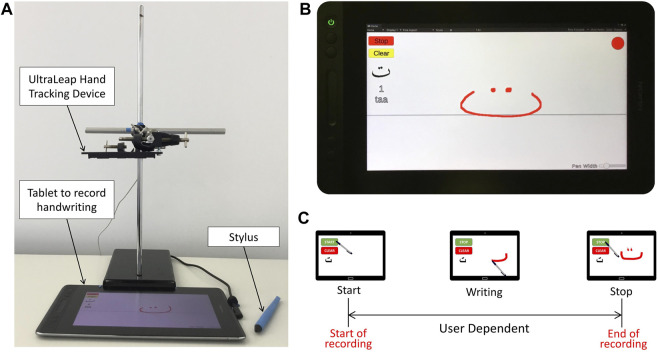
**(A)** Experimental setup, **(B)** User interface, **(C)** Timeline of the single trial.

A snapshot of the experimental setup is shown in Figure 1A. The proposed setup consists of a HUION GT-116 tablet that is equipped with a pen-like stylus, and an Ultraleap Stereo IR 170 hand movement tracker. As shown in Figure 1, the hand tracking device is attached to a rigid stand in a position where the writer’s hand movement can be reliably tracked. Note that the setup is portable to facilitate data collection at different sites.

### 2.2 Experimental task and protocol

The handwriting of all the 28 letters of the Arabic alphabet in standard form are included in the study. The experts are asked to write each letter separately with their own speed, initiating the process of recording by pressing the “Start” button on the screen and then “Stop” to submit the data or “Clear” to cancel the current recording and repeat the task for the same letter (Figure 1B). The 28 letters are presented in alphabetical order, starting from the first letter ا (“alif”) to the last letter ي (“yaa”). This is repeated 20 times for every letter so we have a total of 560 samples of handwriting to every expert. Experts are encouraged to take a few minutes break whenever needed.

### 2.3 Participants

Two groups of experts are recruited for this study. All experts were native Arabic speakers with no known disorders in handwriting. Five handwriting experts (3 female, 2 male, ages 35–55 years) are recruited to complete the handwriting tasks and 20 Arabic teaching experts (10 male, 10 female, ages 35–55 years) are recruited to complete the online survey. Experts were selected based on the following criteria: 1) more than 10 years of experience in teaching Arabic handwriting and 2) currently work in official (statutory work) or extra-official settings (non-statutory work). Additional inclusion criteria are defined for the group who are recruited to complete the handwriting tasks: 1) available for in-person meeting (for recording the handwriting tasks) and 2) no known disorders in handwriting. The 5 handwriting experts did not rate the difficulty level of the handwriting tasks. Note that the study was conducted in compliance with the Declaration of Helsinki, following its norms and regulations, and with an authorized protocol by the New York University Abu Dhabi Institutional Review Board (IRB: #HRPP-2020–12).

### 2.4 Data collection

A total of 117 spatio-temporal and kinematic features representing the point on the multidimensional skill path are collected (see Table 1). The recordings from the tablet and the hand tracking device are synchronized to concatenate the two feature sets into one. The data were sampled with the rate of 25 Hz.

**TABLE 1:** The 117 features collected during the experiment along with timestamp and hand ID (“Right”/“Left”).

Feature type	Feature name
Stylus kinematics	$\left(x,y,z\right)$ coordinates of tooltip’s trajectory (*z* = const), pressure, azimuth, altitude, proximity
Hand kinematics	Fingers: the distal ends $\left(x,y,z\right)$ coordinates of the bone for distal, intermediate, proximal, metacarpal for all 5 fingers and additionally a proximal end of the metacarpal bone for Index, Middle, Ring and Pinky fingers
	$\left(x,y,z\right)$ coordinates of palm center
	$\left(x,y,z\right)$ coordinates of hand pinch position (thumb and index if they are pinched)
	$\left(x,y,z\right)$ coordinates of hand predicted pinch position
	$\left(x,y,z\right)$ coordinates of hand wrist position
	$\left(x,y,z\right)$ coordinates of elbow position (estimated if not in view)
	$\left(x,y,z\right)$ coordinates of hand arm center (midpoint of the bone)
	$\left(v_{x},v_{y},v_{z}\right)$ palm speed
	$\left(n_{x},n_{y},n_{z}\right)$ hand palm normal
	$\left(r_{x},r_{y},r_{z},r_{w}\right)$ hand rotation
	Palm width, pitch, yaw, roll
	Hand pinch strength, hand pinch distance
	Hand grab angle
	Hand arm length (length of the bone)
	Hand arm width (average width of flesh around the bone)

### 2.5 Data preprocessing

Due to the differences in the experts’ writing speed, the collected raw data represent a multivariate time series of 117 features of different duration, ranging from 15 time points to 234 time points. The data with duration less then 24 time points are excluded from the analysis, because it requires at least 1 s to accomplish the writing task and everything below this threshold represents a partially written letter. Also, the first and last two time points are trimmed to remove any noise during pen’s approaching to and distancing from the tablet surface. To make the data uniform, the features × time points tensors are padded by zeros to create 117 × 234 matrices. All the variables are normalized before feeding them to the classification model.

#### 2.5.1 Multiclass single-label classification

**FIGURE 2:**
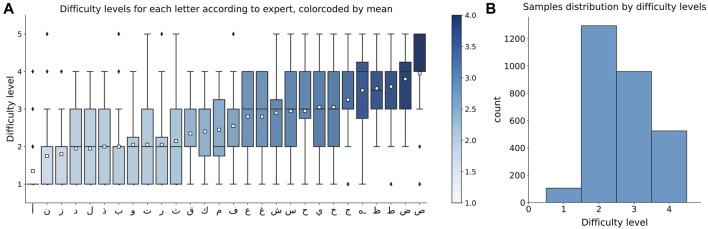
**(A)** Distribution of difficulty levels evaluated by experts for each letter, color coded by mean values for each letter, **(B)** Distribution of samples, which difficulty level is labeled by averaging and rounding the experts’ evaluations.

The traditional multiclass single-label classification is concerned with training a machine learning model on a set of samples, each having a unique class label from a set of disjoint class labels either binary (two classes) or multiclass (more than two classes) (Sorower, 2010). In this work the labels for multiclass single-label classification were obtained by extracting the mean values from the distribution of the difficulty levels evaluated by experts for each letter and rounding it to the nearest integer (Figure 2, A). It is clear that most of the letters were evaluated as of difficulty level 2 and 3 by the majority of experts. The experts were usually quite reluctant to mark the letter of difficulty level 5, therefore the taken means are always below 5 and therefore difficulty level 5 was excluded from consideration. The apparent imbalance in letters distribution by difficulty levels (Figure 2B) prompted us to seek another labeling approach that reflects each expert’s opinion regarding the difficulty of a given letter while concurrently preserving the overall distribution of difficulty estimates. For this reason, the multiclass-multilabel classification approach has been adopted alongside multiclass single-label classification.

### 2.5.2 Multiclass-multilabel classification

In contrast to a single-label classification where class labels are mutually exclusive, the multilabel classification (or multi-output classification) is a generalization of multiclass single-label classification that deals with multiple mutually non-exclusive repeated classes or “labels.” A given training set consists of *n* samples $S=\left(x_{i},y_{i}\right)$ , *i* = 1, … , *n*, $x_{i}=\left(x_{i}^{1},\ldots ,x_{i}^{f}\right)\in X$ , $y_{i}=\left(y_{i}^{1},\ldots ,y_{i}^{l}\right)\in Y$ , where X is the instance space, Y is the label vector space, *f* is the number of features and *l* is the number of labels in a label vector. The samples are independent, identically distributed and are randomly drawn from an unknown distribution *D*. The multilabel learning aims to produce a multilabel classifier h:X→Y that optimizes specific evaluation function (i.e., loss function) (modified from (Zhang and Zhou, 2007)). In this work a vector $y_{i}=\left(y_{i}^{1},\ldots ,y_{i}^{l}\right)$ of labels is referred to “label vector”.

Emerged from automatic text-categorization for medical diagnosis, the multilabel classification can be accomplished using Artificial Neural Networks that allow to specify the number of outputs in the output layer. The applications of multilabel classification can be found in music/movie categorization, where a single melody/movie can belong to different genres, or in semantic scene classification, where a picture can be categorized under more than one conceptual class at the same time (e.g., sunsets and beaches) (Tsoumakas and Katakis, 2007; Sorower, 2010).

To create the labels vectors for multiclass-multilabel classification, we define a labels vector of size 1 × 5 for each letter. Each label in the labels vector refer to the number of experts who evaluated the letter as a particular difficulty level. Each label can range from 0 to 20 (since there were 20 experts in overall). For example, for letter ا “alif” the target label vector is $\left[16,2,1,1,0\right]$ , meaning that sixteen experts out of twenty evaluated it as of level 1, two experts as level 2, one expert as level 3 and as level 4 each and no one as level 5.

### 2.6 Feature selection: Shapley values

Shapley values, first introduced in the context of Game Theory (Shapley, 1953), are becoming more popular for interpreting the contribution of each feature in the prediction process of a trained ML model (Fryer et al., 2021). The idea behind Shapley values is to evaluate the influence of each feature on the outcome of the model by sequentially replacing each of the features, one at a time, with random uniformly-distributed values. A machine learning model is then repeatedly trained on the samples from the training set with the introduced randomly populated feature. Shapley values are obtained by calculating the difference between the outcome of the model considering the random feature and the outcome of the model considering the original feature. This is done on all instances of the validation set. Then, Shapley values are averaged across the samples of the validation set to get the overall influence of each feature. The model is iteratively retrained on the different subsets of the features, replacing a different feature with the random values each time. At the end, the distribution of averaged Shapley values is obtained, allowing to test the hypothesis if the replaced feature is more important than the introduced random feature.

In general, Shapley value of feature with index $f\in F=\left\{1,\ldots ,d\right\}$ , where *F* is a set of all features’ indices, can be defined as a weighted average over all marginal contributions *M*_*f*_(*S*) of *f*, where the marginal contribution of *f* to submodel *S* ⊂ *F* is defined as a difference in evaluation when *f* is added to the submodel:

$$M_{f}\left(S\right)=C\left(S\cup \left\{f\right\}\right)-C\left(S\right)$$ (1)

where *C* is an evaluation function.

Thus, the Shapley value *ϕ*_*f*_ of feature of index *f* is

$$\phi_{f}=\underset{S\in 2^{F\backslash \left\{f\right\}}}{\sum }\omega \left(S\right)M_{f}\left(S\right),$$ (2)

and the weights are $\omega \left(S\right)=\frac{|S|!\left(|F|-|S|-1\right)!}{\left|F\right|}!$ (Fryer et al., 2021).

## 3 Multiclass single-label classification

### 3.1 Architecture

**FIGURE 3:**
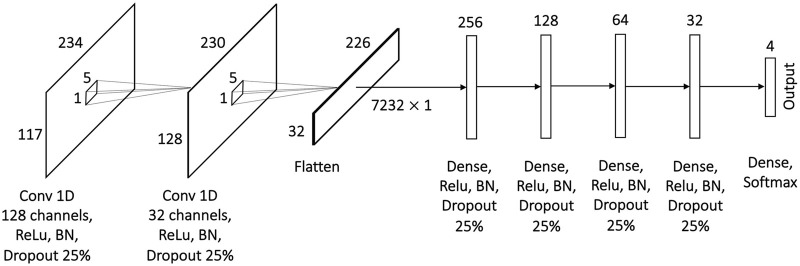
Proposed architecture of the 1D CNN model for multiclass single-label classification with convolution across time.

The 1D CNN model for multiclass single-label classification contains two 1D convolution layers for data preprocessing and five fully connected layers for learning (Figure 3). The first convolution layer has 128 channels and accepts a matrix of 117 features and 234 time points as an input. The convolution is performed along the time dimension using 1 × 5 kernel. The resulting 128 × 230 matrix is passed to the input of the second 1D convolution layer with the same 1 × 5 kernel sliding along the time dimension. The output from the second 1D convolution layer is flattened and passed to the dense layers with 256, 128, 64, 32 and finally 4 neurons. To stabilize the learning process and to prevent overfitting, the batch normalization and dropout of 25% were applied after each layer. The Rectified Linear Unit (ReLU) activation was used in all layers except the last one (output layer) that uses Softmax as an activation function. The model was trained on 5000 epochs with Adaptive Moment Estimation (Adam) optimizer and sparse categorical crossentropy loss. The optimal learning rate of 10^–4^ was found empirically. Due to the significant imbalance in the number of samples for average difficulty levels 1 and 4 in comparison to difficulty levels 2 and 3 (see Figure 2) weights are used in the loss function to penalize higher for wrong prediction of levels 1 and 4 during the model training.

### 3.2 Results

#### 3.2.1 Model evaluation

**FIGURE 4:**
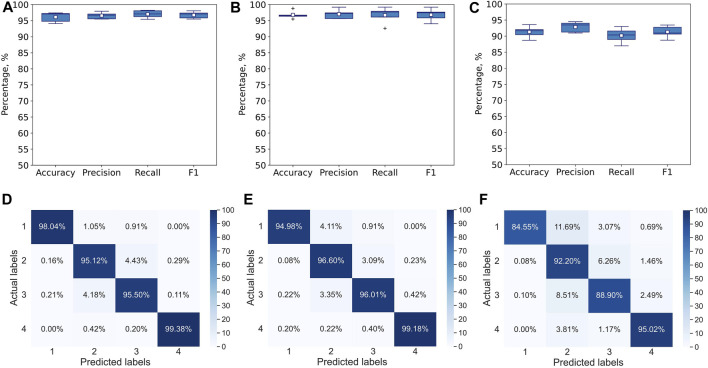
Performance of multiclass single-label 1D CNN model with convolution across time after 5000 epochs for 5-fold CV. The metrics are averaged over all 4 classes for each of the folds. The model is trained and tested with **(A)** all 117 features, **(B)** Seven stylus kinematics features, **(C)** 110 hand kinematics features. Corresponding confusion matrices averaged over 5 folds: **(D)** for all 117 features, **(E)** for 7 stylus kinematics features, **(F)** for 110 hand kinematics features.

**FIGURE 5:**
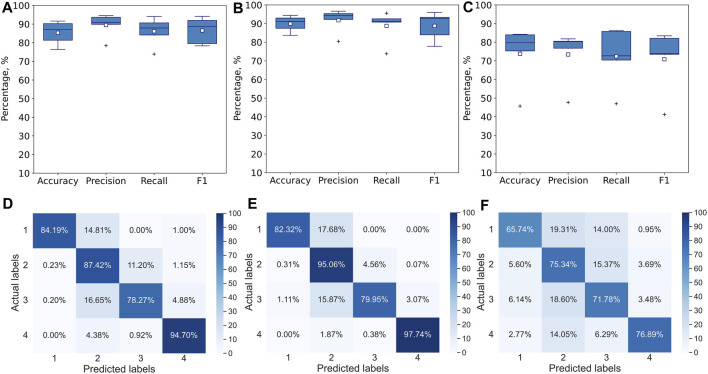
Performance of multiclass single-label 1D CNN model with convolution across time after 5000 epochs for leave-one-expert-out CV. The metrics are averaged over all 4 classes for each of the folds. Model has been trained and tested with **(A)** all 117 features, **(B)** Seven stylus kinematics features, **(C)** 110 hand kinematics features. Corresponding confusion matrices averaged over 5 folds: **(D)** for all 117 features, **(E)** for 7 stylus kinematics features, **(F)** for 110 hand kinematics features.

The 1D CNN model is evaluated using 5-fold cross-validation (Figure 4) and leave-one-expert-out cross-validation (Figure 5) in terms of accuracy, precision, recall and F1-score. To understand the effect of hand-kinematics related features, the model is trained on three different datasets. One dataset contained all 117 features, and the other two contained only the 7 stylus kinematics features and the 110 hand kinematics features, respectively. Training on all three datasets is run for 5000 epochs and evaluated with 5-fold and leave-one-expert-out cross-validation.

The 1D CNN model for multiclass single-label classification achieves accuracy of 96%, recall of 97% and precision of 97% after 5-fold cross-validation with all 117 features (Figures 4A, D). With the stylus kinematics features, the model achieves highest average accuracy, recall and precision were all 97% (Figures 4B, E). With the hand kinematics features, the average accuracy drops to 91%, the recall to 90%, and precision to 93% 4, C, F).

A similar analysis has been performed with leave-one-expert-out cross-validation. As expected, the performance of the model drops. The skill of handwriting is individualized and varies from expert to expert and since during the leave-one-expert-out cross-validation the model is trained with data from any of the four experts and tested on data from the fifth’s, it learns handwriting features common to these four experts but fails to capture some of the individualized aspects of the handwriting of the fifth expert. For example, if the fifth expert is left-handed while the remaining four experts are right-handed, the model will learn only right-handed features determining difficulty levels (e.g., the tilt of the pen) and will not be able to infer the difficulty level from the same features of the left-handed individual, if they differ significantly from right-handed participants. Similar to 5-fold cross validation, the highest accuracy of 90% with recall of 89% and precision of 92% has been achieved on the dataset with stylus kinematics features (Figures 5B, E), while the lowest one of 74% with recall of 72% and precision of 73% is observed on the dataset with the hand tracking features (Figures 5C, F). Despite the noticeable drop in performance, the model is still capable to use some general features to infer the difficulty level of the handwriting task.

#### 3.2.2 Feature analysis

**FIGURE 6:**
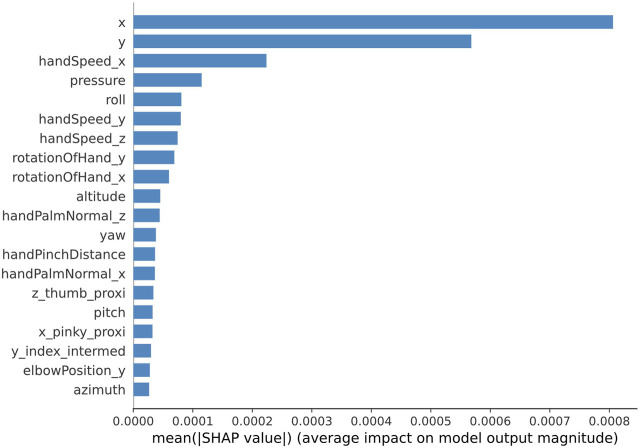
Shapley values of the top 20 features learned by the 1D CNN model trained for 5000 epochs (single run) for multiclass single-label classification.

To determine which features are considered as significant for classifying handwriting tasks into four difficulty levels, Shapley values are calculated. The single-run accuracy of the trained model was 98%. Shapley values are calculated using randomly selected 100 instances from the training set as the background distribution and randomly selected 100 instances from the testing set for validation. For each of the 100 test instances, the calculated Shapley values are selected only for the classes that correspond to the true labels. The obtained Shapley values are averaged across 100 validation instances. The 20 most prominent features for the multiclass single-label classification are shown in Figure 6. These results demonstrate that positional coordinates of the stylus and pressure data as well as roll and hand speed from hand tracking device are significant features for evaluating the difficulty level of the handwriting task.

## 4 Multiclass-multilabel classification

### 4.1 Architecture

**FIGURE 7:**
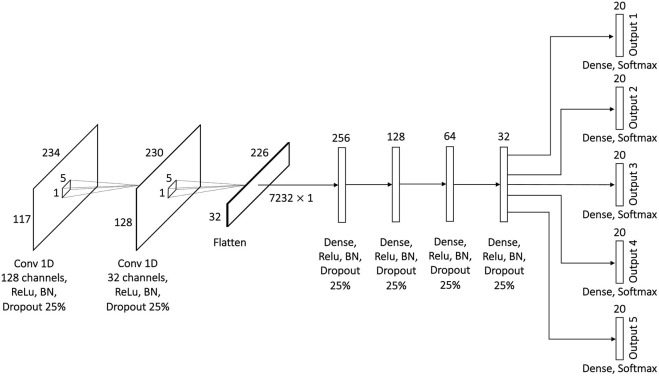
Proposed architecture of the 1D CNN model for multilabel classification with convolution across time.

The developed model for multiclass-multilabel classification has architecture similar to the multiclass single-label model. In other words, it also contains two 1D convolution layers for data preprocessing and five fully connected layers for learning (Figure 7). The input to the first convolution layer of 128 channels is a matrix of 117 features and 234 time points. The convolution is performed along the time dimension using 1 × 5 kernel. The resulting 128 × 230 matrix is passed to the input of the second 1D convolution layer with the same 1 × 5 kernel sliding along the time dimension. The output from the second 1D convolution layer is flattened and passed to the dense layers with 256, 128, 64 and 32 neurons. The only difference from the multiclass single-label model’s architecture is that in the multilabel model the output of the penultimate layer with 32 neurons is passed to 5 parallel dense layers with 20 neurons and Softmax activation. Each one of the 5 output layers represents a difficulty level. A single output layer produces a number between 1 and 20 which represents the number of experts who estimated the letter as of a corresponding difficulty level. The outputs from each of these layers later combined to form a label vector of 5 elements. To stabilize the learning process and to prevent overfitting the batch normalization and dropout of 25% were applied after each layer. The Rectified Linear Unit (ReLU) activation used in all layers except the output layers. The model was trained on 5000 epochs with Adaptive Moment Estimation (Adam) optimizer and categorical crossentropy loss. The optimal learning rate of 10^–4^ was found empirically.

The network for multilabel classification outputs a label vector of 5 elements, whose index-position corresponds to the given difficulty level and values corresponds to the number of experts evaluating the letter with a particular difficulty level. We consider the performance metrics such as accuracy, precision, recall and F1-score separately for each of the difficulty level across 5 folds. The accuracy is averaged across 5 difficulty levels and 5 folds.

### 4.2 Results

#### 4.2.1 Model evaluation

The 1D CNN model was evaluated using 5-fold cross-validation and leave-one-expert-out cross-validation. Apart from common metrics such as accuracy, precision, recall and F1-score Godbole and Sarawagi (2004), averaged over all samples for a given fold/expert and for each difficulty level, the Exact Match Ratio (EMR) (Sorower, 2010) was used to give additional insight into the model’s performance in case of the multilabel classification. The EMR score shows how many elements (labels) in predicted label vector equal to the labels in actual label vector (both labels’ values and positions must coincide). The requirements were to predict both the label value and its position in the label vector. Formally speaking, if we denote

$$y_{i}=\left(y_{i}^{1},\ldots ,y_{i}^{l}\right)\in Y$$

–an actual label vector, where Y is the label vector space, *i* = 1, *…* , *n*, *n*–number of samples, *l* is the number of labels in a label vector, and

$${\hat{y}}_{i}=\left({\hat{y}}_{i}^{1},\ldots ,{\hat{y}}_{i}^{l}\right)\in \hat{Y}$$

–a predicted label vector of *n*th sample, where $\hat{Y}$ is the predicted label vector space, then the exact match ratio is defined as

$$EMR=\frac{1}{n}\overset{n}{\underset{i=1}{\sum }}I\left(y_{i}={\hat{y}}_{i}\right),$$ (3)

Where I is the indicator function. formula 3 can be extended if we are interested in exact match of vectors with any number of correctly predicted labels. For example, in case of *l* − 1 correctly predicted labels:

$$\begin{array}{ll} {EMR}^{l-1} & =\frac{1}{n}\overset{n}{\underset{i=1}{\sum }}I\left(y_{i}^{j}={\left.{\hat{y}}_{i}^{j}\right|}_{j=2}^{l}\wedge y_{1}^{j}\neq {\hat{y}}_{1}^{j}\right)+\ldots \\ & \;+\frac{1}{n}\overset{n}{\underset{i=1}{\sum }}I\left(y_{i}^{j}={\left.{\hat{y}}_{i}^{j}\right|}_{j=1}^{l-1}\wedge y_{l}^{j}\neq {\hat{y}}_{l}^{j}\right), \end{array}$$ (4)

The accuracy was calculated as a ratio of the number of correctly predicted labels to the total number of labels for a given instance:

$$A=\frac{1}{n}\overset{n}{\underset{i=1}{\sum }}\frac{\left|y_{i}\cap {\hat{y}}_{i}\right|}{\left|y_{i}\cup {\hat{y}}_{i}\right|}$$ (5)

The precision was calculated as a ratio of the number of correctly predicted labels to the total number of actual labels averaged over all instances:

$$P=\frac{1}{n}\overset{n}{\underset{i=1}{\sum }}\frac{\left|y_{i}\cap {\hat{y}}_{i}\right|}{\left|y_{i}\right|}$$ (6)

The recall was calculated as a ratio of the number of correctly predicted labels to the total number of predicted labels averaged over all instances

$$R=\frac{1}{n}\overset{n}{\underset{i=1}{\sum }}\frac{\left|y_{i}\cap {\hat{y}}_{i}\right|}{\left|{\hat{y}}_{i}\right|}$$ (7)

The F1 score was calculated as a harmonic mean of the precision and recall:

$$F_{1}=\frac{1}{n}\overset{n}{\underset{i=1}{\sum }}\frac{2|y_{i}\cap {\hat{y}}_{i}|}{\left|y_{i}|+|{\hat{y}}_{i}\right|}$$ (8)

**FIGURE 8:**
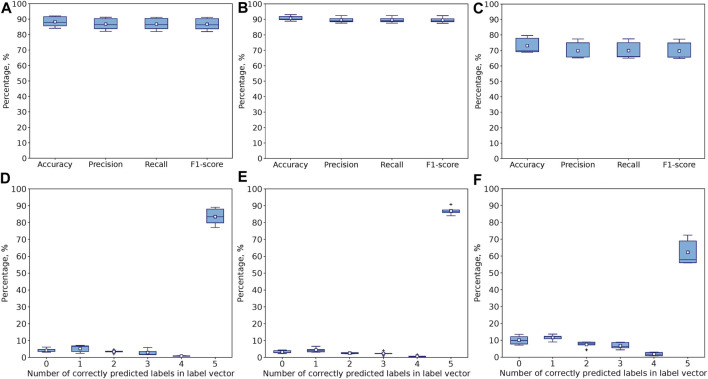
Performance of 1D CNN model after 5000 epochs for 5-fold CV with **(A)** all 117, **(B)** Seven stylus kinematics, and **(C)** 110 hand kinematics features. Percentage of samples with correctly predicted number of label vector’s elements (both positions and values) for **(D)** all 117, **(E)** Seven stylus kinematics, and **(F)** 110 hand kinematics features. Corresponds to Exact Match Ratio in the case of 5 labels.

The metrics were averaged over all folds. For example, Figure 8D. Shows the performance of the model across 5 folds with all 117 features by comparing how many label vectors have at least one common element (both value and position are the same), two common elements, and so on. For 5 common elements, depending on the fold, the Exact Match Ratio varies between 77.1% and 89.1%. Notice that 3.1%–6.1% of all samples were predicted incorrectly (none of the elements of predicted and actual label vectors coincide).

**FIGURE 9:**
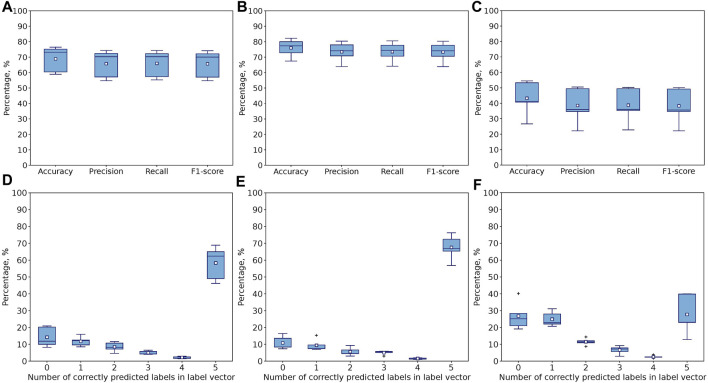
Performance of 1D CNN model after 5000 epochs for leave-one-expert-out CV with **(A)** all 117 features, **(B)** Seven stylus kinematics, and **(C)** 110 hand kinematics features. Percentage of samples with correctly predicted number of label vector’s elements (both positions and values) for **(D)** all 117, **(E)** Seven stylus kinematics, and **(F)** 110 hand kinematics features. Corresponds to Exact Match Ratio in the case of 5 labels.

The results of model’s evaluation are demonstrated in Figure 8 (5-fold cross-validation) and Figure 9 (leave-one-expert-out cross-validation) with accuracy, precision, recall and F1 averaged over all samples for each difficulty level on the upper row (A., B., C) and the accuracy of predicting the exact value and position of the element corresponding to the difficulty level in the label vector on the lower row (D., E., F.). The model trained for 5000 epochs performs better for 5-fold cross-validation rather than leave-one-expert-out cross-validation, because the latter excludes the unique features, attributable to the experts from training, while the 5-fold cross-validation ensures that samples of all experts are equally present both in training and testing sets.

The prevalence of metrics obtained for the model trained and evaluated on the stylus kinematics features over hand kinematics features in the multiclass single-label classification is also observed for the multilabel case. It is more prominent for the leave-one-expert-out cross-validation (Figure 9C) but also true for the 5-fold cross-validation (Figure 8), where the use of 110 hand-tracking features results in increased variation in metrics across folds. For example, the average accuracy score for 5-fold cross validation drops from 91% to 73% when switching from stylus kinematics features to hand kinematics features. For leave-one-expert-out cross validation this gap is more significant (the average accuracy drops from 76% to 43%).

**FIGURE 10:**
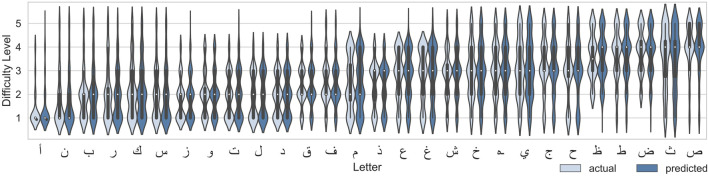
Distribution of actual levels and predicted levels with 1D CNN model after 5000 epochs (single run) for each letter.

It is also interesting to evaluate whether the 1D CNN model trained for 5000 epochs on the dataset containing all 117 features is capable of capturing the distribution of the number of experts (the values of each element) in the label vector. Figure 10 shows the violin plots for each letter comparing the actual and predicted distributions of number of experts who assigned the corresponding difficulty level to that letter. At the first glance, it is easy to notice that most of the distribution shapes are predicted correctly. Moreover, some letters have a similar distribution, for example, most of the experts evaluated ا “alif” and ن “nuun” as belonging to level 1, while the letters ز (“zaay”), and و (“waaw”) were evaluated by the majority of experts as level 1 and 2 and as level 4 by only a few experts. In general the distributions are reconstructed correctly, with noticeable similarities in distributions of different letters (ب (“baa”) and ރ (“raa”), or ح (“Haa”), حـ (“khaa”), and ي (“yaa”)). In some cases as ا “alif” most of the experts agree on the difficulty level, while for other cases like م (“miim”) there is almost uniform distribution of expert evaluations across the five levels; meaning that the experts cannot agree on how challenging it is to write this letter.

**FIGURE 11:**
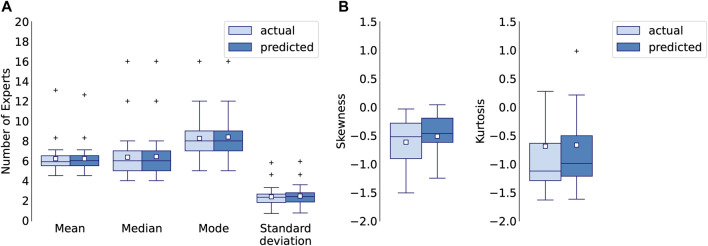
**(A)** Mean, median, mode, standard deviation, **(B)** skewness and kurtosis of actual and predicted distributions for 1D CNN model after 5000 epochs (single run), calculated for each letter.

The 1D CNN model for multiclass-multilabel classification accurately predicts the parameters of the distribution of the number of experts across difficulty levels. Figure 11 A. compares mean, median and mode of the predicted and actual distributions and 11 B. shows the standard deviation, kurtosis and skewness of the predicted and actual distributions for each letter.

#### 4.2.2 Feature analysis

**FIGURE 12:**
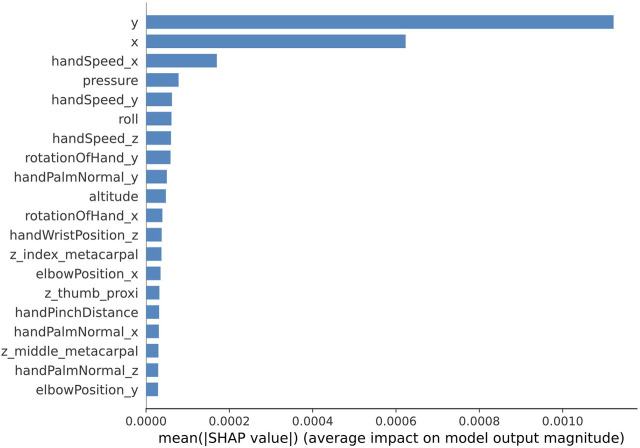
Shapley values of the top 20 features learned by the 1D CNN model trained for 5000 epochs (single run) for multiclass-multilabel classification.

Similar analysis with Shapley values was conducted for the multiclass-multilabel classification. The average accuracy of the model across all classes and all labels was 89.8%, the average precision 90.2%, the average recall and F1 score were 90% and 89.8% correspondingly, and the Exact Match Ratio was 85.7%. Shapley values were calculated for every element of the label vector in the same way as shown previously with the single-label classification. Finally, the obtained Shapley values were averaged across 100 validation instances and then average along 5 elements of the label vector. Figure 12B. Shows the most prominent 20 features. These results mostly coincide with the results obtained for the multiclass single-label classification. It appears that the handwriting stylus positional coordinates, hand speed and roll from the hand tracking device and also the pressure data are the most prominent for evaluating the difficulty level of the handwriting task.

## 5 Discussion

In general, the 1D CNN model demonstrates better performance for 5-fold cross-validation in comparison to the leave-one-expert-out cross-validation, both for single-label and multilabel classification. The leave-one-expert-out cross-validation shows the ability of the model to rate the task included in the training set but executed differently by a left-out expert in comparison to the similar task in the training set. The model will perform well if it does not use the individual features of expert’s sensorimotor skill for ranking the difficulty level of the handwriting task. Therefore, the lower performance of the model in leave-one-expert-out cross validation can be indicative of model’s ability to focus on the features describing the individuality of expert’s sensorimotor skill in the evaluation of the handwriting tasks. Similarly, during the 5-fold cross-validation for every fold the model is trained on 80% of the tasks coming from all the experts and therefore, it learns common features defining the difficulty level of the task. In this case, the high performance of the model might indicate its ability to pick up the features associated with the experts’ generic skills.

Another common observation between the single-label and multilabel models is that both models perform the best when trained based on the stylus kinematics. On the other hand, relying on the hand kinematics (i.e. 110 features obtained from the handtracking system) results in a relatively compromised performance, due to the high dimensionality of feature space that leads to the model overfitting. Nonetheless, it is interesting to observe that it is possible to predict the difficulty level of a handwriting task with an accuracy that is considerably above the chance level by relying on hand kinematics only without any direct information about the trajectory of the task.

Explainability analysis with Shapley values provides further insight on which features contribute the most towards the classification of the difficulty level. The previously mentioned conclusion on the prevalence of handwriting trajectory features is consolidated by the results of Shapley values, where it has been demonstrated that the importance of the handwriting trajectory features, collected from the stylus, prevail over the hand kinematics features. The top 3 handwriting trajectory features are geometric coordinates of the stylus-tip (*x* and *y*) and the pressure while the top three hand kinematics features include hand velocity components and the roll, for single-label classification. For multilabel classification the most prominent are handwriting trajectory features as stylus tip coordinates, pressure, height (altitude) of the stylus tilt. These features prevail over the top most prominent hand kinematics features including all three components of hand velocity, and roll.

As mentioned before, the motivation for multiclass-multilabel classification is twofold: 1) The majority of the alphabets are ranked as difficulty levels 2 and 3, leading to highly unbalanced ratings as shown in Figure 2. 2) Alphabets with similar means (i.e., they have the same labels in multiclass single-label classification) could have very different underlying distributions. Both motivations are linked to the fact that the difficulty level of a handwriting task is subjective; and thus, alphabets are more characterized with distributions of difficulty levels rather that a single level. Figure 10 gives an insight on how well the multilabel model performed in classifying the distribution of difficulty level compared to the actual one. It can be observed that different letter have different distribution patterns. For example, some letters have uni-modal distribution where experts somewhat agree on a difficulty level (easy, intermediate, or hard) while other letters are bimodal where experts have split opinions on the difficulty level of the letter (mode1: easy, mode2: hard). Other letters show a uniform distribution where experts have uniformly split over the different difficulty levels. These major differences in the distributions demonstrate the need of multilabel classification.

## 6 Conclusion

The main purpose of this work is to offer a system for automatic evaluation of the difficulty level of handwriting task. The traditional Machine Learning approach for a single-label classification relies on the difficulty level defined as an average of the distribution of difficulty levels evaluated by experts, rounded to the nearest integer. While this approach can give an idea of the overall difficulty of the handwriting task, the knowledge of the distribution of experts’ evaluations allow to understand the extend of subjectivity of experts’ judgment. A highly biased distribution with smaller standard deviations will indicate that most of the experts are agree on the difficulty level of the task, while the low values of standard deviation of multimodal distribution indicates the disagreement among the experts. The proposed multilabel model automatically determines the distribution of experts’ opinions about the difficulty level of the task from the spatio-temporal features of the hand without asking the experts to evaluate it. This information will help to select the task that corresponds to the skill level of the writer. The model can be used in adaptive handwriting teaching systems that adjust the difficulty level of handwriting task based on the evaluation of the handwriting quality of a learner.

As per future work, we plan to adapt the model to evaluate the difficulty level of other handwriting tasks than Arabic letters. For instance, the model can be re-trained/modified to evaluate the difficulty level of handwriting in other languages or even abstract shapes. Furthermore, the collected data was acquired on a digit tablet. It would be interesting to evaluate the model with another acquisition system that allows writing on a physical paper (e.g., Wacom Bamboo) or on devices that simulate paper (e.g., reMarkable).

## Data Availability

The datasets presented in this study can be found in online repositories. The names of the repository/repositories and accession number(s) can be found below: https://osf.io/kq86d/.
